# Aeroelastic Behavior of 3D-Printed Tapered Polylactic Acid Plates Under Subsonic Flow Conditions

**DOI:** 10.3390/ma18051127

**Published:** 2025-03-02

**Authors:** Mirko Dinulović, Mato Perić, Dragi Stamenković, Aleksandar Bengin, Vuk Adžić, Marta Trninić

**Affiliations:** 1 Faculty of Mechanical Engineering, University of Belgrade, Kraljice Marije 16, 11000 Belgrade, Serbia; mdinulovic@mas.bg.ac.rs (M.D.); abengin@mas.bg.ac.rs (A.B.); vadzic@mas.bg.ac.rs (V.A.); 2 Department of Mechatronics, University North, Trg dr. Žarka Dolinara 1, 48000 Koprivnica, Croatia; 3 College of Applied Studies Aviation Academy, Bulevar vojvode Bojovica 2, 11158 Belgrade, Serbia; stamenkovic.d@vakademija.edu.rs; 4 The Academy of Applied Studies Polytechnic, Katarine Ambrozić 3, 11000 Belgrade, Serbia; mtrninic@atssb.edu.rs

**Keywords:** PLA, plate stability, 3D printing, flutter, wind tunnel, subsonic flow

## Abstract

This research investigates the aeroelastic stability of tapered polylactic acid (PLA) plates produced through fused deposition modeling (FDM) under low-Mach-number airflow conditions. While the static properties of 3D-printed structural components for drones, unmanned aerial vehicles (UAVs), and unmanned aircraft systems (UAS) have been thoroughly explored, their dynamic behavior, especially flutter, has been less studied. This study applies a binary flutter model to thin PLA plates, and the analytically predicted flutter speeds are compared with experimental data from wind-tunnel tests. The strong agreement between theoretical predictions and experimental results confirms the validity of the proposed dynamic aeroelastic analysis approach. This methodology provides valuable insights into designing aerodynamic lifting and stabilizing surfaces for UAS applications.

## 1. Introduction

Additive manufacturing includes a range of techniques in which parts are built layer by layer using digital files, typically generated from CAD systems or 3D object scanners. Unlike traditional methods where a part is cut or milled from a solid block, additive manufacturing creates components by gradually adding material in successive layers. In recent years, these techniques have continued to evolve and have become in focus for researchers and original equipment manufacturers (OEMs).

The development of additive manufacturing technology has greatly influenced various manufacturing processes, streamlined numerous designs, and significantly reduced the time required for prototyping. In addition, additive manufacturing techniques have changed assembly methods and significantly simplified the construction of complex structural parts. With the improvement of additive production technology, 3D-printed components have moved from the field of research and development to conventional manufacturing and production lines. The aerospace industry was among the early adopters of additive manufacturing in both the design and production phases. Because the aerospace industry adheres to some of the most stringent industry standards, requiring parts to function flawlessly under extreme conditions for extended periods of time, it becomes a critical area for the application of this innovative technology.

Both commercial and military aerospace applications demand flight-worthy components. Metals, ceramics, thermoplastics, and composites are being explored for their potential use in aerospace and their suitability for additive manufacturing processes [1].

One of the additive manufacturing techniques is fused deposition modeling (FDM), which falls under the material extrusion category of 3D printing technology. This process involves building parts layer by layer by extruding heated plastic filament through a nozzle. A wide range of polymers are available in filament form and are suitable for 3D printing. For general-purpose applications, the most commonly used polymers include polylactic acid (PLA), acrylonitrile butadiene styrene (ABS), polyethylene terephthalate (PET), nylon, thermoplastic elastomer (TPE), and polycarbonate. Filaments such as PLA/carbon blends, polypropylene, acetal, polymethyl methacrylate (PMMA), and fluorene polyester (FPE) are also available for more advanced applications.

Polylactic acid, commonly known as PLA, is one of the most widely used materials in 3D printing. For most extrusion-based 3D printers, PLA is the standard filament choice because it requires lower printing temperatures and often does not need a heated bed. Additionally, PLA is cost-effective and can be utilized across a broad range of industrial applications. From an environmental perspective, PLA stands out as one of the most eco-friendly materials [2,3,4].

The advantages of PLA include its low cost, stiffness, relatively good strength, excellent dimensional accuracy, and long shelf life. However, it also has some drawbacks, such as low heat resistance, the need for cooling fans during printing, brittleness that can cause the filament to break, and sensitivity to UV light.

In recent years, fused deposition modeling (FDM) techniques using thermoplastic filament materials have become ever more popular, mostly in the design and prototyping phases of drones, unmanned aerial vehicles (UAVs), and unmanned aircraft systems (UASs) [5]. Many researchers have devoted their efforts to studying the physical and mechanical properties of FDM-produced parts for flying vehicles [6], including their tensile strengths and failure modes [7]. Additionally, the effect of printing process parameters on the quality and performance of manufactured parts has been widely investigated [8,9].

This paper investigates the aeroelastic stability of tapered plates manufactured from polylactic acid (PLA), using the advanced PLA filament 3D printing technology, subjected to axial subsonic flow. Due to fluid–structure interactions, the plates deform under aerodynamic loading, which alters the distribution of aerodynamic forces and further influences these deformations. This interdependent feedback mechanism results in an aeroelastic phenomenon known as flutter—a self-excited and often destructive oscillation where the energy is extracted from the airflow.

The tapered plate analyzed in this paper could correspond to a wing or stabilizer either with or without control surfaces. The interaction between a cantilevered elastic plate in a uniform axial flow is a canonical fluid–structure interaction problem. It is well known that this system may exhibit flutter instability under low subsonic flow. Once the freestream velocity exceeds a critical value, the structure transitions to large and violent limit cycle oscillations. Flutter is a potentially damaging dynamic aeroelastic phenomenon where aerodynamic forces interact with the natural vibration modes, causing periodic motion of a structure and leading to system instability. If the system lacks sufficient aerodynamic damping, the vibration amplitude increases, eventually leading to structural failure [10,11].

The structure of the paper is as follows: Section 2 introduces the aeroelastic tapered-plate model, with quasi-steady flutter and unsteady flutter analysis discussed in Section 2.1 and Section 2.2, respectively. Section 3 details the preparation of wind-tunnel samples and model predictions, including density measurement (Section 3.1), flexural modulus of elasticity measurement (Section 3.2), modal characteristics (Section 3.3), and analysis (Section 3.4). The wind-tunnel experiment is presented in Section 4, followed by concluding remarks in Section 5.

## 2. Aeroelastic Tapered-Plate Model

The prime interest of this research is the coupled-mode flutter of 3D-printed tapered PLA plates exposed to subsonic flows. This phenomenon arises when two eigenmodes of fluid–structure interaction coincide, leading to high-amplitude structural oscillations and resulting in dynamic instability. These high amplitudes induce significant strains, which, in turn, generate high stresses within the structure, potentially leading to complete structural failure [12,13].

**Figure 1:**
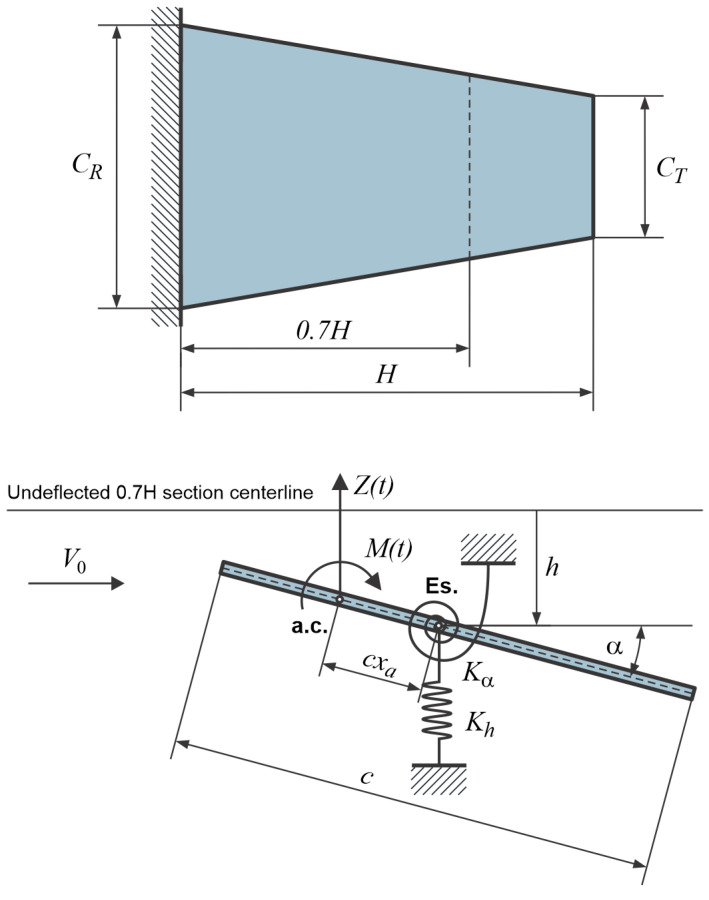
Tapered plate’s geometry, analysis coordinates, and aerodynamic forces on a typical plate section (a.c.—aerodynamic center, $cx_{a}$—relative distance between shear and aerodynamic centers).

To derive the expression for the critical flutter speed, it is assumed that the system being analyzed is clamped at the root plate chord location, allowing the plate to undergo small vertical and angular displacements. It is useful to describe the system’s motion using two generalized coordinates: *h* for plunging motion and α for twisting. The geometry of the tapered thin plate analyzed in this paper is illustrated in Figure 1. Consequently, the system is treated as a two degrees-of-freedom (2 dof) system. For a thin flat plate with a rectangular cross-section, the shear center (center of twist) and the center of gravity are located along a spanwise line at the half-midchord (c/2) position. The aerodynamic center is assumed to be positioned forward of the midchord by a distance of $cx_{a}$. The aerodynamic normal force Z(t) and the aerodynamic moment M(t) act at the aerodynamic center (a.c.).

In flutter analysis, selecting the appropriate aerodynamic theory is crucial. The choice of theory generally depends on the Mach number range within which flutter is anticipated. In this study, both quasi-steady flutter and the more general case of unsteady flutter have been investigated. These two scenarios can be distinguished by evaluating the reduced frequencies (*k*) specific to each case. The reduced frequency represents the ratio of the vibration frequency to a reference frequency, which is defined by the linear flight speed divided by half the chord length [14]. The reduced frequency can be expressed mathematically as follows:

(1) $$k=\frac{\omega \cdot c}{2V_{0}}.$$

In the previous equation, $V_{0}$ represents the linear flight speed, *c* is the plate chord (average) exposed length, and *ω* denotes the frequency of vibration. Small values of the reduced frequency, k<0.1, indicate a slow flapping and twisting motion of the structure relative to the linear flight speed [15]. In this scenario, the problem can be treated as quasi-steady. However, for values of k>0.1, an unsteady analysis is required.

The unsteady flutter analysis is applicable across all ranges of reduced frequencies. This method takes into account the influence of “artificial” structural damping and must be examined using unsteady aerodynamic theories. As a result, the unsteady flutter analysis is more computationally intensive than the quasi-steady analysis. The subsequent paragraphs will discuss both the quasi-steady and unsteady flutter cases.

### 2.1. Quasi-Steady Flutter

Assuming small plate taper and aspect ratios, neglecting pitching moment about the aerodynamic center, the aerodynamic lift force Z(t), used for flutter speed calculation ($V_{F}$), can be expressed as follows:

(2) $$Z\left(t\right)=-\frac{1}{2}C_{L\alpha }\cdot \alpha \left(t\right)\cdot \rho \cdot V_{0}^{2}\cdot c,$$

where $C_{L\alpha }$ is the lift curve slope (with a theoretical value of 2π/rad for a flat plate), α(t) is the angle of attack, and *ρ* is the density of the air at the flight altitude. Since, in this paper, tapered plates are analyzed, the adjustment to the theoretical plate lift curve slope is carried out using known Prandtl–Glauert corrections for compressibility and plate aspect ratio:

(3) $$C_{L\alpha }=\frac{2\pi \cdot A}{2+\sqrt{A^{2}+4-\frac{A^{2}\cdot {V_{0}}^{2}}{{a_{0}}^{2}}}}.$$

In the previous equation, *A* is the plate aspect ratio, and $a_{0}$ is the speed of sound.

Writing the kinetic and potential energy equations for the plate cross typical section model shown in Figure 1, using Lagrange’s approach, the equations of motion can be written in the following form:

(4) $$\begin{array}{c} m\ddot{h}+K_{h}h=-\frac{1}{2}C_{L\alpha }\alpha (t)\rho {V_{0}}^{2}c\\ I_{\alpha }\ddot{\alpha }+K_{\alpha }\alpha =\frac{1}{2}C_{L\alpha }\alpha (t)\rho {V_{0}}^{2}x_{a}c^{2}, \end{array}$$

where *m* is the plate mass (spanwise), $K_{h}$ is the bending stiffness, $I_{\alpha }$ is the mass moment of inertia about the plate midchord line, and $K_{\alpha }$ is the torsional stiffness.

The solution to the previous set of equations is obtained by assuming harmonic motion:

(5) $$\begin{array}{c} h=\bar{h}\cdot sin(\omega t)\\ \alpha =\bar{\alpha }\cdot sin(\omega t) \end{array},$$

where $\bar{h},\;\bar{\alpha }$ represent eigenvectors in plunging and twisting, respectively. In previous equations, ω is the frequency of motion (coupled). Also, the following substitutions are introduced for convenience:

(6) $$\omega_{h}^{2}=\frac{K_{h}}{m},\;\;\omega_{\alpha }^{2}=\frac{K_{\alpha }}{I_{\alpha }},\;\;I_{\alpha }=\frac{mc^{2}r_{\alpha }^{2}}{4},\;\mathrm{and}\;\mu =\frac{4m}{\pi cb^{2}}$$

In the above, $\omega_{h}$ and $\omega_{\alpha }$ are natural frequencies in the bending and twisting of the analyzed system, $r_{\alpha }$ is the radius of gyration about the midchord, and *μ* is the mass ratio of the dimensionless plate to the airstreams. After making the substitutions presented in (6) and incorporating second derivatives of assumed harmonic motions (5) into equations of motion (3), the characteristic equation is obtained in the following form:

(7) $$\omega^{4}+\omega^{2}\left(\frac{8C_{L}U^{2}x_{a}}{r_{\alpha }^{2}\mu c^{2}\pi }-\omega_{h}^{2}-\omega_{\alpha }^{2}\right)+\left(\omega_{h}^{2}\cdot \omega_{\alpha }^{2}-\frac{\omega_{h}^{2}8C_{L}U^{2}x_{a}}{r_{\alpha }^{2}\mu c^{2}\pi }\right)=0.$$

The aforementioned fourth-order characteristic equation yields four solutions. The analyzed system is considered to be undamped, with no mass imbalance, and the shear center is located at the midchord of the characteristic section. In this case, two of the solutions are positive and are presented in Equation (8).

(8) $$\begin{array}{l} \omega_{1}=\omega_{h}\;\\ \omega_{2}=\sqrt{\frac{\mu \pi \left(\omega_{\alpha }^{2}r_{\alpha }^{2}\mu c^{2}\pi -8C_{L\alpha }{V_{0}}^{2}x_{a}\right)}{cr_{\alpha }\mu \pi }} \end{array}$$

Flutter occurs when $\omega_{1}=\omega_{2}$. The convergence of the two frequencies signifies the boundary between damped and undamped vibration, characterized by a neutrally stable oscillation. Hence, the quasi-steady flutter velocity can be calculated as follows:

(9) $$V_{F}=U(\omega_{1}=\omega_{2})=\sqrt{\frac{\pi c^{2}r_{\alpha }^{2}\mu \left(\omega_{\alpha }^{2}-\omega_{h}^{2}\right)}{8C_{L\alpha }x_{a}}}$$

Provided that all conditions are satisfied as mentioned earlier and that the natural frequencies in bending and twisting are known, the above equation (Equation (9)) is valid for estimating the critical flutter speed of tapered thin plates. When using this approach, it is advisable to verify its validity by calculating the value of reduced frequency at flutter, followed by the verification that the quasi-steady conditions are met (k<0.10). The reduced frequency criterion at flutter is given as

(10) $$k_{F}=\frac{\omega_{h}\cdot c}{2\cdot V_{F}}<0.10.$$

The natural frequencies of the thin plate (in bending and twisting) required in Equation (9) can be obtained either experimentally or by performing the modal analysis for the tapered plate.

### 2.2. Unsteady Flutter Analysis

In cases where the criteria in Equation (10) are not satisfied, an unsteady flutter analysis must be performed. Unsteady flutter analysis includes the effects of ‘artificial’ damping and requires that the aerodynamic forces be expressed using unsteady aerodynamics.

To account for damping in the unsteady analysis, a dissipation function is used and is given in the following form:

(11) $$D=\frac{1}{2}\frac{g_{h}K_{h}{\dot{h}}^{2}}{\omega }+\frac{1}{2}\frac{g_{\alpha }K_{\alpha }{\dot{\alpha }}^{2}}{\omega }$$

In the previous equation, terms $g_{h}K_{h}\dot{h}$ and $g_{\alpha }K_{\alpha }{\dot{\alpha }}^{2}$ represent damping forces in bending and twisting. Using the dissipation function and assuming system motion in the form $h=h_{0}\cdot e^{i\omega t},\;\alpha =\alpha_{0}\cdot e^{i\left(\omega t+\theta \right)}$, where *θ* is the phase angle between bending and twisting (bending amplitude is ahead of twisting), the equations of motion for unsteady thin-plate flutter become the following:

(12) $$\begin{array}{l} \ddot{h}+\frac{S_{k}}{m}\ddot{\alpha }+g_{h}\frac{\omega_{h}^{2}}{\omega }\dot{h}+\omega_{h}^{2}h=-\frac{Z\left(t\right)}{m}\\ \ddot{\alpha }+\frac{S_{k}}{I_{\alpha }}\ddot{h}+g_{\alpha }\frac{\omega_{\alpha }^{2}}{\omega }\dot{\alpha }+\omega_{\alpha }^{2}\alpha =\frac{M\left(t\right)}{I_{\alpha }} \end{array}$$

In the previous equation, $S_{k}$ is a static moment of inertia of the characteristic section of the plate analyzed.

One of the closed-form analytical solutions for unsteady airflow in relation to flat plates is proposed by Theodorsen and is based on linear potential flow. Normal force Z(t) and moment M(t), in the previous equation (Equation (12)), can be expressed as a function of reduced frequency. Plunging and pitching motions at a.c. are expressed as complex quantities, through oscillatory lift due to pitch, oscillatory lift due to plunge, and also oscillatory moment due to pitch motion [15]. Moment due to plunging motion, according to Theodorsen’s unsteady theory, is considered constant. Normal lift and aerodynamic moment, according to Theodorsen, are given in the following form:

(13) $$\begin{array}{l} z(t{)}_{\alpha }=\frac{1}{2}-i\left(\frac{1}{k}\right)\cdot \left[1+2\left(F+iG\right)\right]-2{\left(\frac{1}{k}\right)}^{2}\left(F+iG\right)\\ z(t{)}_{h}=1-2i\left(\frac{1}{k}\right)\cdot \left(F+iG\right) \end{array}$$

(14) $$\begin{array}{l} M(t{)}_{\alpha }=\frac{3}{8}-i\left(\frac{1}{k}\right)\\ M(t{)}_{h}=\frac{1}{2} \end{array}$$

Equation (13) contains terms *F* and *G*, which represent in-phase and out-of-phase components of Theodorsen’s circulation function, C(k), for the assumed harmonic plate motion. The circulation function is a function of the reduced frequency (*k*). Functions *F* and *G* are given in the following form [16]:

(15) $$\begin{array}{l} C(k)=F(k)+iG(k)\\ C(k)=1-\frac{0.165}{1-\frac{0.0455}{k}i}-\frac{0.335}{1-\frac{0.30}{k}i}\;k\leq 0.5\\ C(k)=1-\frac{0.165}{1-\frac{0.0410}{k}i}-\frac{0.335}{1-\frac{0.32}{k}i}\;k>0.5 \end{array}$$

The unsteady flutter solution requires iterative calculations. Typically, structural damping versus linear velocity is graphically represented for both bending and torsion modes. The process begins with an initial estimate of the flutter reduced frequency, often obtained from the quasi-steady flutter case due to its lower computational cost. Using this initial value, the Theodorsen aerodynamic functions needed in Equation (12) are computed. The equations are then iteratively solved to obtain pairs of damping and velocity for specific values of the reduced frequency (*k*). Once a sufficient number of data points is generated, graphs plotting artificial structural damping against velocity for each mode are constructed. These graphs must contain enough data points to ensure accuracy. The flutter velocity ($V_{F}$) is identified at the intersection of the graph with the velocity axis, where damping becomes zero, indicating the onset of instability.

## 3. Sample Preparation for Wind-Tunnel Testing and Model Prediction

**Figure 2:**
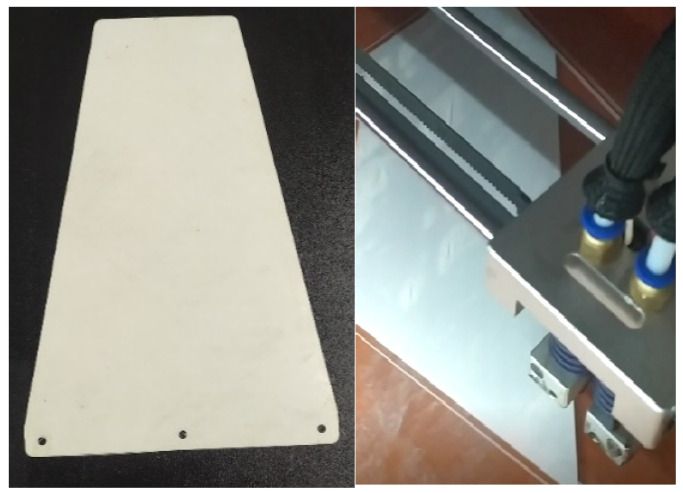
PLA 3D-printed plate.

The initial model analysis began with the selection of the material for the tapered plate. One of the primary objectives of this research was to assess the applicability of PLA material for potential use in UAVs and analyze its aeroelastic stability. Consequently, the tapered plate was manufactured using FDM technology on a 3D printer with PLA filament [17]. The thin tapered plate used in the analysis and experimental wind-tunnel testing is shown in Figure 2.

The mechanical behavior of certain materials, particularly polymers and composites, exhibits nonlinear properties under varying loading conditions. To accurately capture these properties, experimental techniques such as tensile, compressive, and shear testing are commonly used in addition to advanced constitutive modeling. For 3D-printed polymers such as PLA, methods such as digital image correlation (DIC), dynamic mechanical analysis (DMA), and rheological testing provide insights into their strain rate dependence and viscoelastic behavior. These approaches enable more precise characterization, which is essential for reliable structural analysis and simulation.

The set of the technological parameters used in the 3D printing process is summarized in Table 1.

The mechanical characteristics and dimensions of the tapered plate are summarized in Table 2.

### 3.1. Density Measurement

The density of the manufactured part was determined experimentally in accordance with ASTM D792, Standard Method A. A rectangular specimen (165 × 20 mm) was cut from the PLA 3D-printed plate. The specimen was first weighed in air and then weighed while immersed in distilled water at 23° C.

### 3.2. Flexural Modulus of Elasticity Measurement

**Figure 3:**
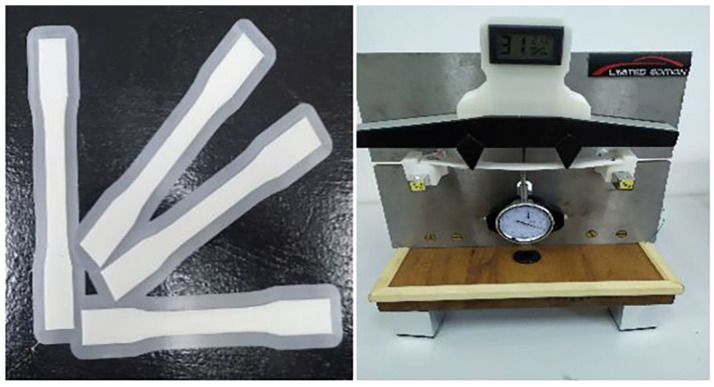
Four-point bending machine for thin-walled structures.

Flexural tests were performed on the same specimens used for density determination in accordance with ASTM D6272. The tests were conducted using a four-point bending machine for thin-walled structures (Figure 3).

Using this approach, Young’s modulus of elasticity was experimentally measured, showing good agreement with the results reported in the literature. The specimens for this experiment were printed on an Ender 5 Pro printer using 1.75 mm PLA filament. The test specimens were printed at 0°, 30°, 45°, and 90°, and Young’s modulus was obtained experimentally using a four-point bending test machine.

The in-plane shear modulus of elasticity ($G_{12}$) was calculated using Equation (16), which represents the in-plane elastic coefficient transformation equation, as given in [18]. Assuming that the values for $E_{1}$, $E_{2}$, and $E_{45}$ are known (experimentally obtained in this paper), the in-plane shear modulus of the PLA-printed specimen can be calculated using the following equation:

(16) $$\frac{1}{E_{45}}=\frac{1}{4}\left(\frac{1}{E_{1}}+\frac{1}{E_{2}}\right)\left(\frac{1}{2}-\frac{\gamma_{12}}{2}\right)+\frac{1}{4G_{12}}$$

It is worth noting that the Poisson ratios (minor and major) are assumed to be equal, with values of $\gamma_{12}=\gamma_{21}=0.3$ [19,20,21].

Accurate values for all material elastic coefficients are required, since they are used in subsequent modal analysis, which highly impacts the calculation of flutter velocity (Equations (9) and (12)).

### 3.3. Modal Characteristics

The natural frequencies of the plates in bending and twisting were experimentally determined through impact testing on specimens designated for wind-tunnel testing.

In experimental modal analysis, structures are typically excited using electrodynamic or servo-hydraulic shakers, controlled by a signal generator and power amplifier. However, for thin-walled structures such as the PLA plates analyzed in this study, a more practical excitation method is an impact hammer equipped with a piezoelectric force transducer. The impact hammer applies a consistent force, which is initially unknown, over a range of frequencies [22].

To determine the relevant frequency range for the tapered PLA plates, finite-element modal analysis was conducted for the first two modes (bending and twisting). The tapered plate was discretized using plate elements based on Kirchhoff’s thin-plate theory, and the first ten normal modes were calculated under a fixed root edge ($C_{R}$) boundary condition. This boundary condition was chosen due to its relevance in UAVs, drones, and similar applications, where the tapered PLA plate functions as a stabilizing fin or lifting surface, clamped to the fuselage.

For the given plate dimensions, the first ten normal modes were found to lie within the 2–105 Hz range. Notably, the first two modes correspond to those required in Equation (9) for quasi-steady flutter analysis and Equation (12) for unsteady flutter analysis.

Modal analysis using the finite-element approach was performed using the commercial software MSC Nastran 2019. For the analyzed plate, modal extraction was based on the Lanczos algorithm [23].

In this particular problem, the Lanczos algorithm was chosen because it is the most efficient method for medium-to-large structural problems. It precisely computes eigenvalues and eigenvectors, has a performance advantage over other methods, does not miss roots, and offers reliable computational efficiency.

**Figure 4:**
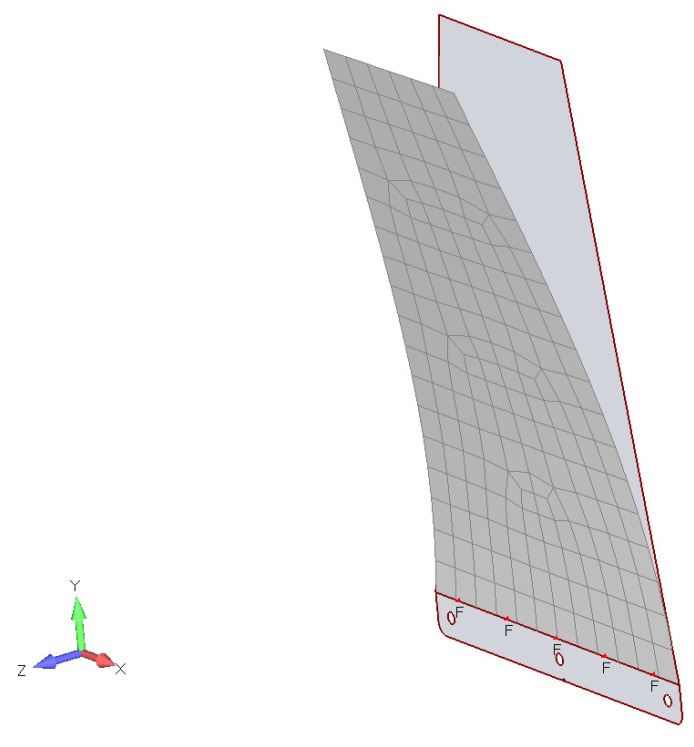
PLA tapered plate’s modal characteristics (mode 1, $\omega_{h}=2.65\;\mathrm{Hz}$).

**Figure 5:**
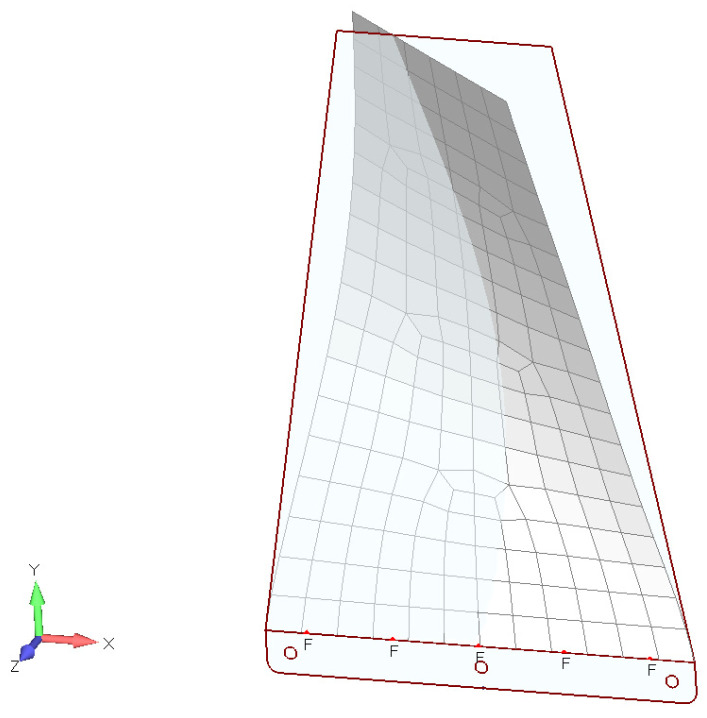
PLA tapered plate’s modal characteristics (mode 2, $\omega_{\alpha }=11.57\;\mathrm{Hz}$).

The material properties of the 3D-printed PLA, such as the density and flexural modulus of elasticity, were obtained experimentally, as described earlier [24,25]. The results of the finite-element modal analysis (first two modes) are presented in Figure 4 and Figure 5:

The identification of the frequency range for normal modes, obtained using the finite-element approach, also enabled the correct selection of the impact hammer head material (metal, rubber, or plastic). This choice is crucial, as it significantly influences the width of the input pulse (bandwidth), as recommended by the OEM for experimental modal analysis.

**Figure 6:**
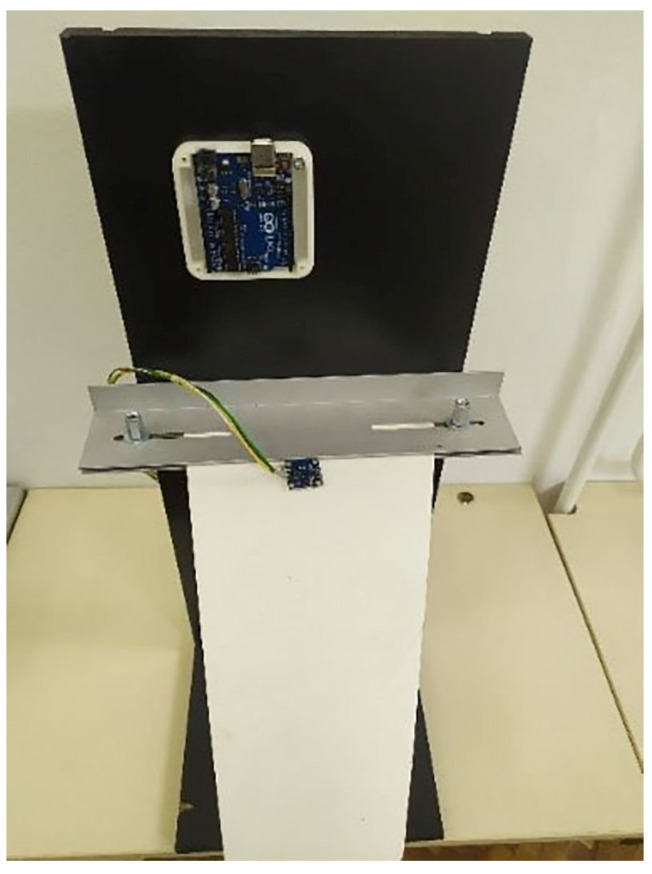
Fixture for a clamped PLA tapered plate for experimental modal analysis.

Experimental modal analysis was performed using the Model 2302-100 modal hammer by Endevco (OEM). An aluminum hammer tip, supplied by the manufacturer, was used in this experiment. The experimental fixture for the analyzed PLA plate clamped at the root chord is shown in Figure 6.

**Figure 7:**
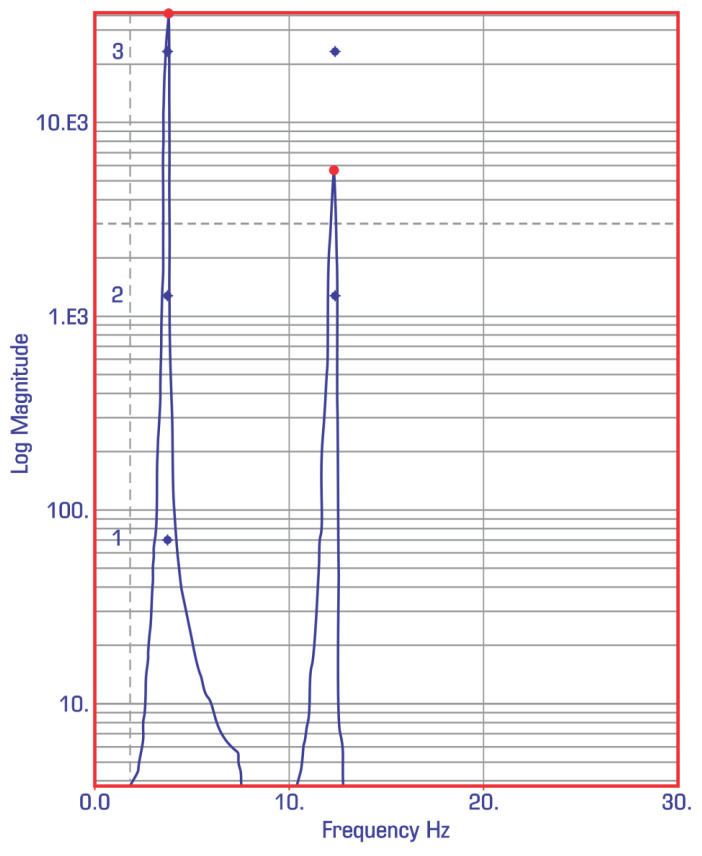
Experimental values for modes 1 and 2 (impact test).

The results obtained from the experimental analysis for the first two modes are presented in Figure 7.

The results of the modal analysis for the first two modes of oscillation, using both methods (numerical and experimental), are summarized in Table 3:

### 3.4. Analysis

Using the approach presented in this paper for the aeroelastic stability of 3D-printed PLA tapered plates, the analyst is able to determine the velocity at which stability loss occurs, i.e., the flutter speed ($V_{F}$), using Equation (12) for the assumed unsteady flutter conditions or Equation (9) for the quasi-steady regime.

As was suggested earlier, the reduced frequency using Equation (10) was calculated in order to determine the flow regime (quasi-steady or unsteady flutter). In this particular case and for the structure’s geometry, modal characteristics, and material mechanical properties, the calculated reduced frequency value revealed that unsteady flutter had to be assumed; hence, the solution of the equations (Equation (10)) was required. Since this was an iterative process, the value of reduced frequency for the quasi-steady flow was used as a starting value in the complete iterative process for the solution of unsteady flutter equations.

**Figure 8:**
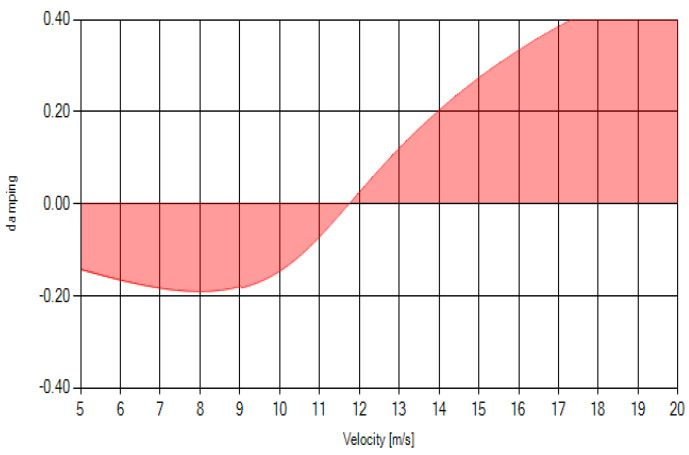
V-g diagram for the 3D-printed PLA tapered plate (dimensions are given in Table 2).

The results obtained are presented in Figure 8, which represents the change in structural damping versus the axial velocity of the flow. For the analyzed geometry and material characteristics, it can be clearly seen from this graph that stability loss occurs at 11.6–11.7 m/s, as it is at that point that the structural damping is close to zero. This point is considered the stability loss point, and the corresponding velocity is regarded as the flutter velocity (flutter speed, $V_{F}$). This value provides valuable information that must be taken into consideration when designing the lifting surfaces of a flying vehicle. Above this velocity (11.6–11.7 m/s in this case, for the analyzed structure), the amplitudes of the surface oscillations increase, and stability loss becomes apparent, possibly leading to complete structural failure. The flutter velocity must be within the flight vehicle’s flight envelope and, as per many standards, reduced by at least 20% to ensure structural integrity.

## 4. Wind-Tunnel Experiment

In order to validate the proposed approach for evaluating the aeroelastic stability of 3D-printed tapered PLA plates, experimental tests were conducted in a subsonic wind tunnel.

**Figure 9:**
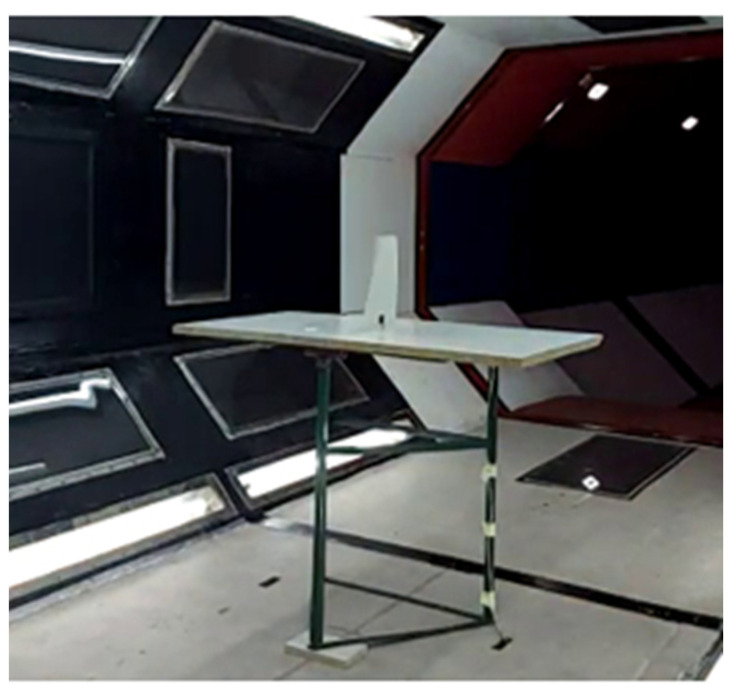
Experimental set-up for wind-tunnel testing.

A specially designed support structure was used to hold the test samples (PLA plates) within the wind tunnel’s test section. The test samples are shown in Figure 2, while the support structure, with a clamped sample at the root chord, is depicted in Figure 9.

To monitor the amplitude of oscillations during testing, accelerometers connected to a DAQ system were mounted on the root chord of the test plate. Airflow velocity was measured using a PCE PFM2 micromanometer with a Pitot tube. The system allowed for the precise control of the wind-tunnel airflow, with velocity adjustments in 0.5 m/s increments and stable airflow regulation.

To ensure repeatability, a total of five samples were manufactured using the same material from the same supplier, produced with identical 3D printing parameters. All samples were tested under the same airflow conditions.

As this study focused on low-Mach-number flows, a subsonic closed-return wind tunnel, capable of generating axial flows up to 50 m/s, was used. The test section of the wind tunnel, with a mounted sample, is shown in the following figure.

The testing procedure was performed as follows: the axial flow was initially set at 5 m/s and, after stabilization, increased in increments of 0.5 m/s until a loss of stability was observed. The amplitudes of oscillations were recorded by the DAQ for all velocities. After the occurrence of stability loss, the velocity was ramped down to the starting flow velocity of 5 m/s with the same increments and stabilization times. This testing procedure was repeated for all the manufactured test samples.

**Figure 10:**
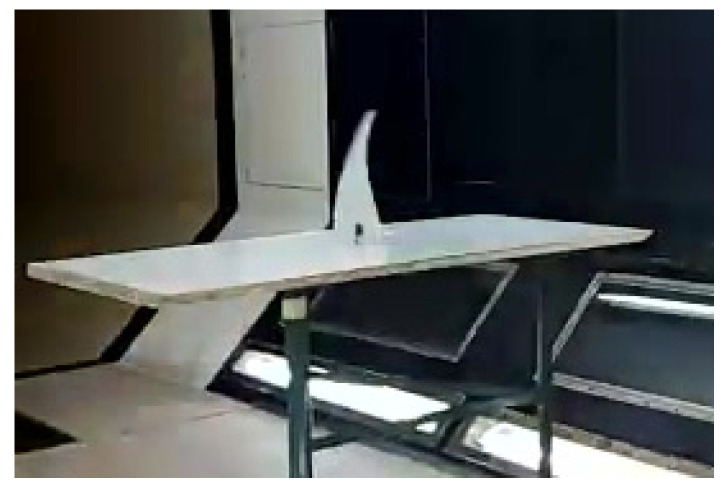
PLA tapered plate’s stability loss in the first part of the oscillation cycle.

**Figure 11:**
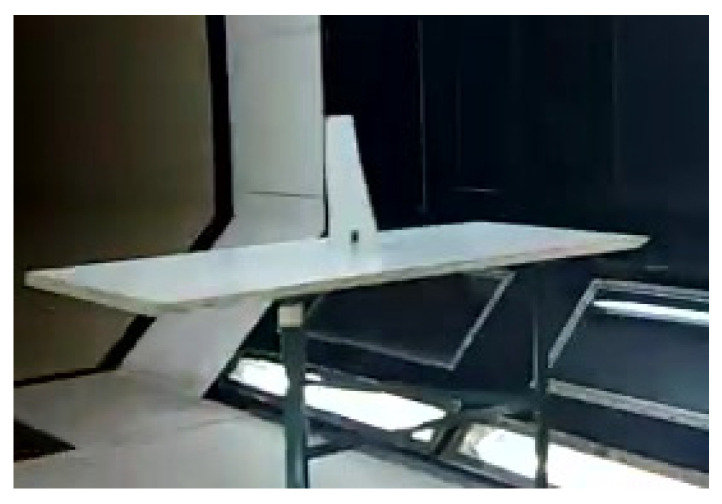
PLA tapered plate’s stability loss in the second part of the oscillation cycle.

**Figure 12:**
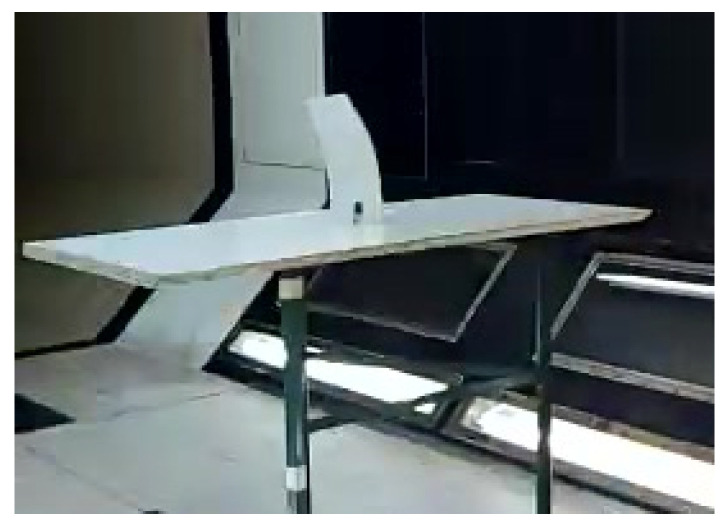
PLA tapered plate’s stability loss in the third part of the oscillation cycle.

The results, showing when aeroelastic stability loss during one oscillation cycle is observed, are presented in Figure 10, Figure 11 and Figure 12.

The test results for each sample tested are summarized in Table 4.

## 5. Conclusions

This study provides valuable insights into the flutter behavior of tapered PLA plates, focusing on the accuracy of the predictive methods and experimental validation. The following key conclusions summarize the main findings from the analysis, which highlight the reliability of the proposed approach, the importance of manufacturing parameters, and recommendations for future research to further enhance the understanding of flutter dynamics in 3D-printed structures.

- The predicted flutter speeds for the tapered PLA plates, based on the known binary aeroelastic model, confirm the aeroelastic theoretical explanation and can be used for flutter speed prediction. The methodology presented in this paper is applicable to PLA tapered plates in all situations where interaction between aerodynamic, inertial, and elastic forces is expected. This conclusion is supported by the fact that the difference between the analytical and experimental results obtained in this paper is within 10%.
- The technological parameters used during the FDM manufacturing process highly influence the mechanical characteristics of the manufactured part and, consequently, the static and dynamic behavior of the structure. These parameters must be handled with caution.
- The recognition of adequate flow conditions is of great importance, and in many situations, unsteady flutter analysis is required.
- All flutter analyses require exact values for modal frequencies, and the existing modal algorithm (Lanczos) for modal extraction provides excellent results, as confirmed by comparing the experimental and numerical results obtained for the structures analyzed in this paper.
- However, the effect of plate taper (the ratio of the tip to root chord) requires further investigation and can be considered one of the recommendations for future work.
- The algorithm (K method) used in this paper to solve the flutter equations, based on the binary model and the assumed Theodorsen unsteady aerodynamic force and moment, is adequate for the problem at hand, as confirmed by comparing the analytical and experimental wind-tunnel results. Another recommendation for future work is to investigate other existing algorithms (such as P-K or PKNL) for solving flutter and analyze their performance (computing time) and accuracy for the types of structures analyzed in this paper.
- By analyzing the material structure of the 3D-printed PLA plates investigated in this study, a certain degree of anisotropy was observed, which required special attention when determining the elastic coefficients.

## Figures and Tables

**Table 1:** Three-dimensional printing parameters for FDM 3D-printed PLA (polylactic acid).

Filament diameter	1.75 mm
Layer thickness	0.2 mm
Extrusion temperature	200° C
Nozzle diameter	0.4 mm
Orientation	Horizontal
Infill orientation	±45°
Printing speed	60 mm/s
Bed temperature	40° C
Number of layers	4

**Table 2:** Tapered plate’s characteristics for FDM 3D-printed PLA (polylactic acid) material.

H	$C_{T}$	$C_{R}$	t
Span	Tip chord	Root chord	Thickness
300 mm	200 mm	125 mm	1 mm
*E*		$\rho_{PLA}$	
Young’s modulus		Density	
1250 MPa		1250 kg/m^3^	
$\omega_{h}$		$\omega_{\alpha }$	
Bending frequency		Torsion frequency	
2.65 Hz		11.57 Hz	

**Table 3:** PLA tapered plate’s modal characteristic for the first two modes.

	$\mathrm{Mode}\;1\;(\omega_{h}$)	$\mathrm{Mode}\;2\;(\omega_{\alpha }$)
FEA	2.65 Hz	11.57 Hz
Experiment	3.17 Hz	12.32 Hz

**Table 4:** Experimental flutter speeds for PLA tapered plates.

Sample No.	V_F_
1	10.5 m/s
2	12 m/s
3	10.5 m/s
4	11 m/s
5	12.5 m/s

## Data Availability

The original contributions presented in this study are included in the article. Further inquiries can be directed to the corresponding author.

## Abbreviations

**Notations**	
*k*	Reduced frequency
*ω*	Frequency of vibration
$V_{0}$	Linear flight speed
*c*	Plate reference chord
$C_{R}$	Root chord
$C_{T}$	Tip chord
*H*	Tapered plate span
*h*	Plunging degree of freedom
*α*	Rotational degree of freedom
M(t)	Aerodynamic moment
Z(t)	Aerodynamic force
*H*	Plate exposed span
$cx_{a}$	Shear center and aerodynamic center distance
a.c.	Aerodynamic center location
*A*	Aspect ratio
$a_{0}$	Speed of sound
$\omega_{h},\;\omega_{\alpha }$	Natural frequencies (in bending and torsion)
$k_{F}$	Reduced frequency (at flutter)
$V_{F}$	Speed at flutter
*g*	Damping
*t*	Plate thickness
$K_{h},\;K_{\alpha }$	Stiffness (bending and torsion)
*μ*	Plate-to-airstream mass ratio
$S_{k}$	Static moment of inertia
$E_{1},\;E_{2}$	Moduli of elasticity (in-fiber and across-fiber directions)
$E_{45}$	Young’s modulus of elasticity in 45° direction
G12	In-plane shear modulus
γ_12,_ γ_21_	Poisson’s ratio (major and minor)
*ρ*	Density of air
$\rho_{PLA}\;$	Density of PLA specimen
